# Case Report: Guanfacine and methylphenidate improved chronic lower back pain in autosomal dominant polycystic kidney disease with comorbid attention deficit hyperactivity disorder and autism spectrum disorder

**DOI:** 10.3389/fped.2023.1283823

**Published:** 2023-11-01

**Authors:** Satoshi Kasahara, Shoichiro Kanda, Miwako Takahashi, Mao Fujioka, Taito Morita, Ko Matsudaira, Naoko Sato, Motoshi Hattori, Toshimitsu Momose, Shin-Ichi Niwa, Kanji Uchida

**Affiliations:** ^1^ Department of Anesthesiology and Pain Relief Center, The University of Tokyo Hospital, Tokyo, Japan; ^2^ Department of Pain Medicine, Fukushima Medical University School of Medicine, Fukushima, Japan; ^3^ Department of Pediatrics, Graduate School of Medicine, The University of Tokyo, Tokyo, Japan; ^4^ Department of Pediatric Nephrology, Tokyo Women’s Medical University, Tokyo, Japan; ^5^ Institute for Quantum Medical Science, National Institutes for Quantum Science and Technology, Chiba, Japan; ^6^ Department of Neuropsychiatry, The University of Tokyo Hospital, Tokyo, Japan; ^7^ Nursing Department, The University of Tokyo Hospital, Tokyo, Japan; ^8^ Institute of Engineering Innovation, School of Engineering, The University of Tokyo, Tokyo, Japan; ^9^ Department of Psychiatry, Aizu Medical Center, Fukushima Medical University, Fukushima, Japan

**Keywords:** autosomal dominant polycystic kidney disease, lower back pain, cystic fibrosis transmembrane conductance regulator, attention deficit hyperactivity disorder, autism spectrum disorder, guanfacine, methylphenidate, cerebral blood flow

## Abstract

Autosomal dominant polycystic kidney disease (ADPKD) is an inherited renal disease characterized by the bilateral development of multiple cysts in the kidneys. Pain management is a clinically important issue, especially because approximately 60% of patients with ADPKD experience chronic pain related to hemorrhage from renal cysts, which significantly reduces their daily life. The cystic fibrosis transmembrane conductance regulator, the molecule responsible for cyst formation in ADPKD, is also the cause of cystic fibrosis. Since attention deficit hyperactivity disorder (ADHD) is known to occur frequently in conjunction with cystic fibrosis, ADPKD may be associated with ADHD. However, to our knowledge, no study has investigated 1) ADHD or autism spectrum disorder (ASD) as comorbidities with ADPKD, 2) the effects of ADHD medications on chronic pain in ADPKD, or 3) cerebral blood flow corresponding to guanfacine (GF) or methylphenidate (MP) treatment for chronic pain. We report the case of a 15-year-old girl with ADPKD, who had chronic back pain associated with ADPKD and had to withdraw from high school because the pain interfered with her daily life. Although she took antihypertensive medications to prevent bleeding, they did not provide adequate blood pressure control. The patient was referred to a child psychiatrist and diagnosed with ASD; however, the pain did not improve. Subsequently, she was referred to our pain center. The diagnosis of ADHD was confirmed and treatment with ADHD medications was initiated. Monotherapy with MP, atomoxetine, and GF resulted in hypertension and hypotension as side effects; however, a combination of MP 18 mg and GF 4 mg provided pain relief and moderate blood pressure control, and the patient was able to go on to college. During the course of treatment, there was an improvement in the distribution of cerebral blood flow in the prefrontal and insular cortices. Confirmation of an ADHD diagnosis comorbid with ASD enabled the use of ADHD medications. The combination of MP and GF improved chronic back pain and high blood pressure due to ADPKD and cerebral blood flow. Screening for ADHD is important in the treatment of ADPKD.

## 1. Introduction

Autosomal dominant polycystic kidney disease (ADPKD), the most common inherited renal disease mainly affecting adults, is characterized by the bilateral development and progressive enlargement of multiple cysts in the kidneys leading to a gradual deterioration of renal function requiring renal dialysis. The estimated prevalence rate is 4.76/10,000 individuals (1, 2). ADPKD is a systemic disease complicated by hypertension, liver cysts, cerebral aneurysms, cystic hemorrhages and infections, and urinary tract stones. Approximately 60% patients with ADPKD experience chronic pain triggered by renal cyst enlargement, bleeding, or infection, which is often difficult to manage and greatly impairs quality of life (QoL) (3). Since up to 30% of patients with ADPKD with chronic pain may require nephrectomy or perhaps liver transplantation, and inadequate pain management might lead to earlier requirement for kidney transplantation or dialysis, pain management is a critical aspect of ADPKD treatment (3).

The mechanism of cyst formation in ADPKD is thought to result from the interaction between abnormal proliferation and apoptosis of tubular epithelial cells caused by an increase in intracellular calcium concentration caused by mutations in the *PKD1* or *PKD2* genes and increased levels of cystic fibrosis transmembrane conductance regulator (CFTR), a transmembrane Cl^−^ channel molecule that promotes the movement and storage of water into the cysts (1).

The CFTR is also the causative agent of cystic fibrosis (CF), an autosomal recessive hereditary disease characterized by the obstruction of exocrine glands throughout the body by viscous secretions and the formation of cysts in the pancreas and other organs (4). Recent studies have shown that attention deficit hyperactivity disorder (ADHD) is frequently comorbid with CF (5–7), with severity of CF symptoms being correlated with the degree of ADHD symptoms (5, 8, 9), and mutations in the CFTR are reportedly associated with ADHD symptoms (8). Studies have also reported that CF and ADHD are caused by ubiquitination-mediated inactivation of the CFTR and dopamine receptor molecules on the cell membrane, respectively (10, 11), with some similarities in their molecular pathogenesis. Hence, ADPKD, in which CFTR dysfunction is responsible for the pathogenesis, may also be associated with ADHD.

However, to the best of our knowledge, no study has reported improvement of chronic pain in ADPKD by ADHD drugs like guanfacine (GF) or methylphenidate (MP), in patients with ADHD or autism spectrum disorder (ASD) being comorbid with ADPKD, together with evaluation of cerebral blood flow corresponding to the course of GF or MP therapy. In this study, we report a case in which GF and MP improved chronic pain associated with ADPKD, ADHD, and ASD, in addition to improving cerebral blood flow.

## 2. Case description

**Figure 1:**
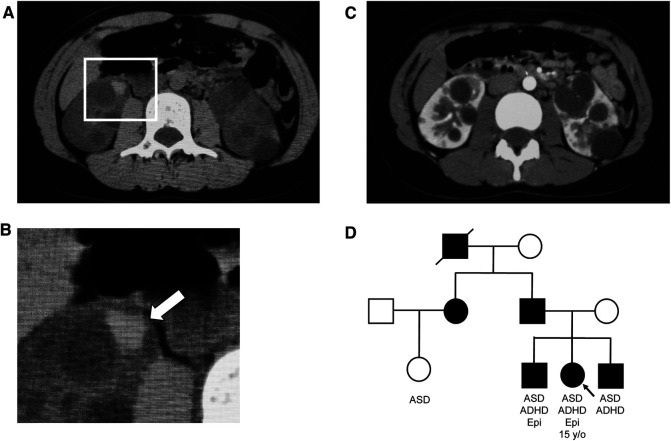
Abdominal computed tomography (CT) images and family tree. (**A**) Simple CT image of the abdomen: Intracystic hemorrhage in the right kidney. (**B**) Enlarged view of the square in (**A**) Arrows indicate intracystic hemorrhage. (**C**) Contrast-enhanced CT image of the abdomen. Numerous cysts of various sizes are present in both kidneys. (**D**) Family tree, with symbols of autosomal dominant polycystic kidney disease-affected individuals painted in black. ADHD, attention deficit hyperactivity disorder; ASD, autism spectrum disorder; Epi, epilepsy.

The patient was a 15-year-old girl (height: 151.7 cm; weight: 42.9 kg; body mass index: 18.6 kg/m^2^). The patient's paternal sister was getting ready to start dialysis, her elder brother and younger brother all had ADPKD, and her paternal cousin, older brother, and younger brother all had ASD. The patient's father was also on dialysis. (Figure 1). Her medical history included childhood-onset fluency disorder and febrile seizures at 5 years of age. She had seizures when she felt exhausted in her second and third grades (2009 and 2010, respectively), and electroencephalography indicated epileptiform discharges that were not treated.

In 2015, the patient underwent computed tomography (CT) at a local clinic on complaining back pain, and was diagnosed with ADPKD and referred to the Department of Nephrology and Pediatrics at Tokyo Women's Medical University Hospital. The pain was persistent and occurred predominantly bilaterally on her back, being exacerbated by exercise. She had difficulty sitting for long periods, which interfered with activities of her daily life. Imaging revealed multiple cysts and microbleeding, but no renal enlargement. Blood and urine examination revealed: white blood cells, 4,850/µl; hemoglobin, 14.2 g/dl; platelets, 160,000/µl; C-reactive protein, 0.02 mg/dl; blood urea nitrogen, 11.8 mg/dl; creatinine, 0.58 mg/dl; Na, 140 mEq/l; K, 3.9 mEq/l; Cl, 105 mEq/l; specific gravity, 1.013; and pH, 5.5; protein, occult blood, and sugar were absent, as were other abnormal findings. Genetic analysis revealed a mutation in *PKD1* (c.11270-3C > G). Blood pressure measured at admission was 123/85 and 142/76 mmHg in the morning and afternoon, respectively. The three most common causes of back pain in ADPKD attributed to the kidneys are hemorrhage, infection, and rapid renal enlargement (12). In this patient, hemorrhage was considered the most likely cause of low back pain, as CT showed hemorrhage in the kidney, tapping pain at the costal vertebral angle, and no evidence of infection or rapid renal enlargement. Considering that cystic hemorrhage associated with hypertension was the cause of pain, candesartan 2 mg/day was prescribed, and conservative treatment with analgesics was initiated. Subsequently, her blood pressure improved slightly to 118/80 mmHg even in the afternoon. However, her symptoms became chronic with repeated improvements and exacerbations, and the doses of analgesics, including acetaminophen 1,800 mg/day, tramadol 75 mg/day, and loxoprofen 60 mg/day, were escalated.

She was bedridden for 2–3 weeks after the onset of pain and required intravenous therapy because she could not eat or drink, and often had to be hospitalized. The patient was academically excellent, securing first position in her grade in secondary school; however, due to her ADPKD symptoms, she was unable to study or participate in school activities and had to discontinue her schooling.

Subsequently, she was referred to a child psychiatrist because of increasing pain, even in the absence of bleeding, whose thorough examination led to suspicion of psychiatric problems. She was diagnosed with ASD but was told that nothing could be done for her pain, and no specific treatment was suggested. Her pain continued to worsen and was difficult to treat. In 2016, she was referred to a psychiatrist at the University of Tokyo Hospital Pain Center.

This study was conducted in accordance with the Declaration of Helsinki and approved by the Research Ethics Committee of Tokyo University Hospital (approval no. 3678). Written informed consent was obtained from the patient and her parents for the publication of any potentially identifiable data included in this article.

## 3. Diagnostic assessment

### 3.1. Initial assessments

On September 8, 2016, the patient visited the outpatient psychiatric clinic at a pain center, and continued visiting once every month, filled out the below-listed questionnaires at each visit, receiving feedback from the psychiatrist regarding the outcomes. Subjective pain intensity was assessed using the numerical rating scale (NRS) (13). A decrease of ≥2 in the NRS was considered either substantial or optimal and designated as the minimum clinically important difference (MCID) (14). Health-related aspects of the QoL were evaluated using the EuroQoL-5 Dimension (15); a score of 0 indicates death and 1.0 indicates perfect health, with an MCID of 0.08 (16). Anxiety and depressive symptoms were assessed using the Hospital Anxiety and Depression Scale-Anxiety/Depression (HADS-A/D) (17); a score of ≥11 was considered as a clinical anxiety/depression (18), with an MCID of 1.5 (19). Pain-related catastrophizing thoughts were assessed using the Pain Catastrophizing Scale (PCS) (20), with a score ≥30 representing above or equal to the 75th percentile in the distribution of scores in patients with chronic pain, with an MCID of 6.48 (16).

**Figure 2:**
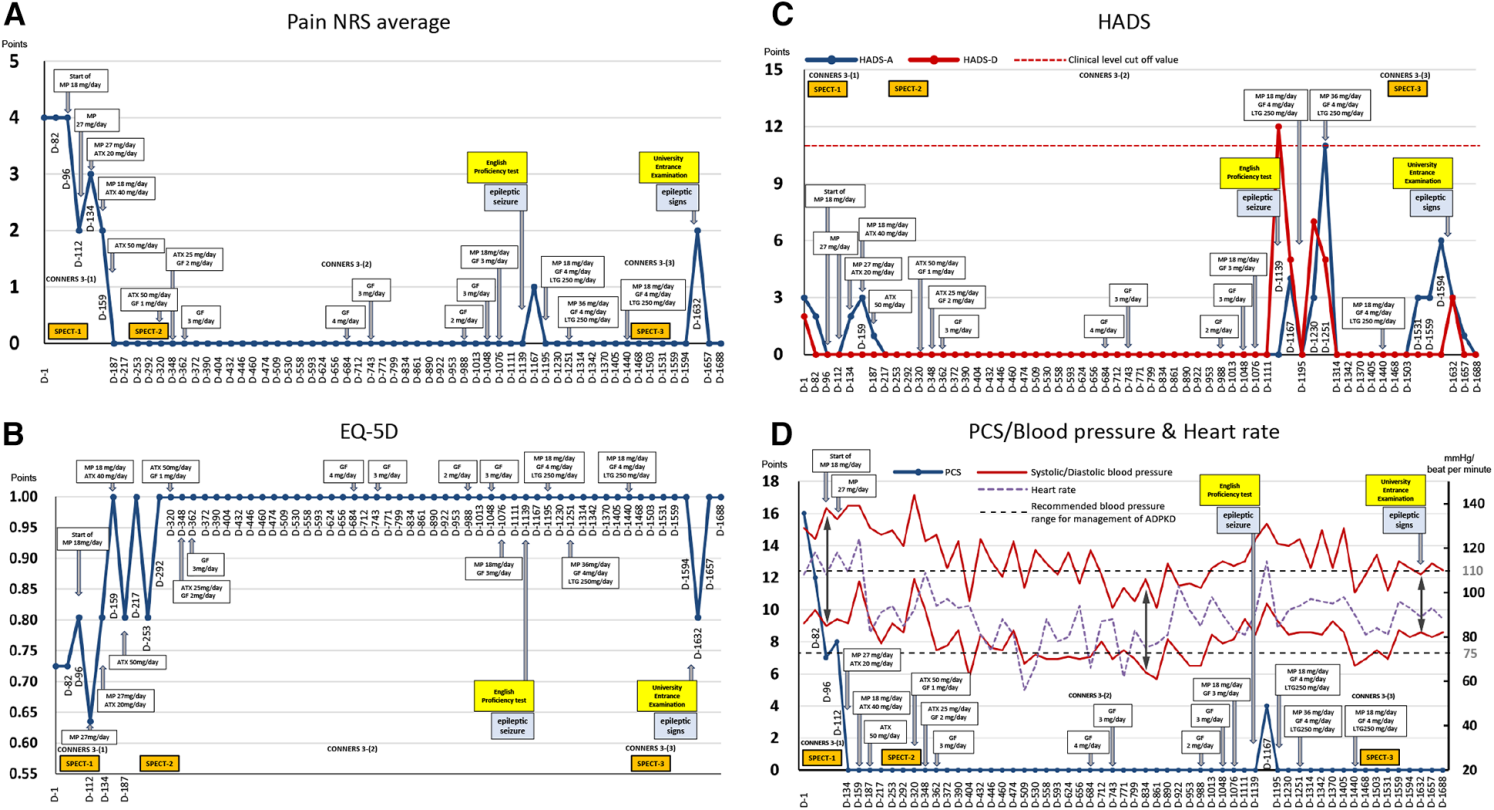
Course of treatment and objective/subjective parameters. (**A**) Average pain numerical rating score (NRS). (**B**) EuroQoL-5 Dimension. (**C**) Hospital Anxiety and Depression Scale. (**D**) Pain Catastrophizing Scale and blood pressure and heart rate. ADPKD, autosomal dominant polycystic kidney disease; ATX, atomoxetine; D, day; EQ-5D, euro QoL 5 Dimension; GF, guanfacine; HADS-A/D, Hospital Anxiety and Depression Scale-Anxiety/Depression; MP, methylphenidate; NRS, numerical rating scale; PCS, Pain Catastrophizing Scale; SPECT, single-photon emission computed tomography.

The patient's average NRS score for back pain at the first visit was 4/10, with EuroQoL-5 Dimension, HADS-A, HADS-D, and PCS scores of 0.725, 3/21, 2/21, and 16/52, respectively (Figure 2).

Experienced psychiatrist SK made a clinical differential diagnosis of psychiatric disorders according to the Diagnostic and Statistical Manual of Mental Disorders, Fifth Edition (DSM-5) (21) criteria. The patient did not experience major depressive episodes with melancholy-type features and was diagnosed with somatic symptom disorder (pain was the primary symptom).

**Figure 3:**
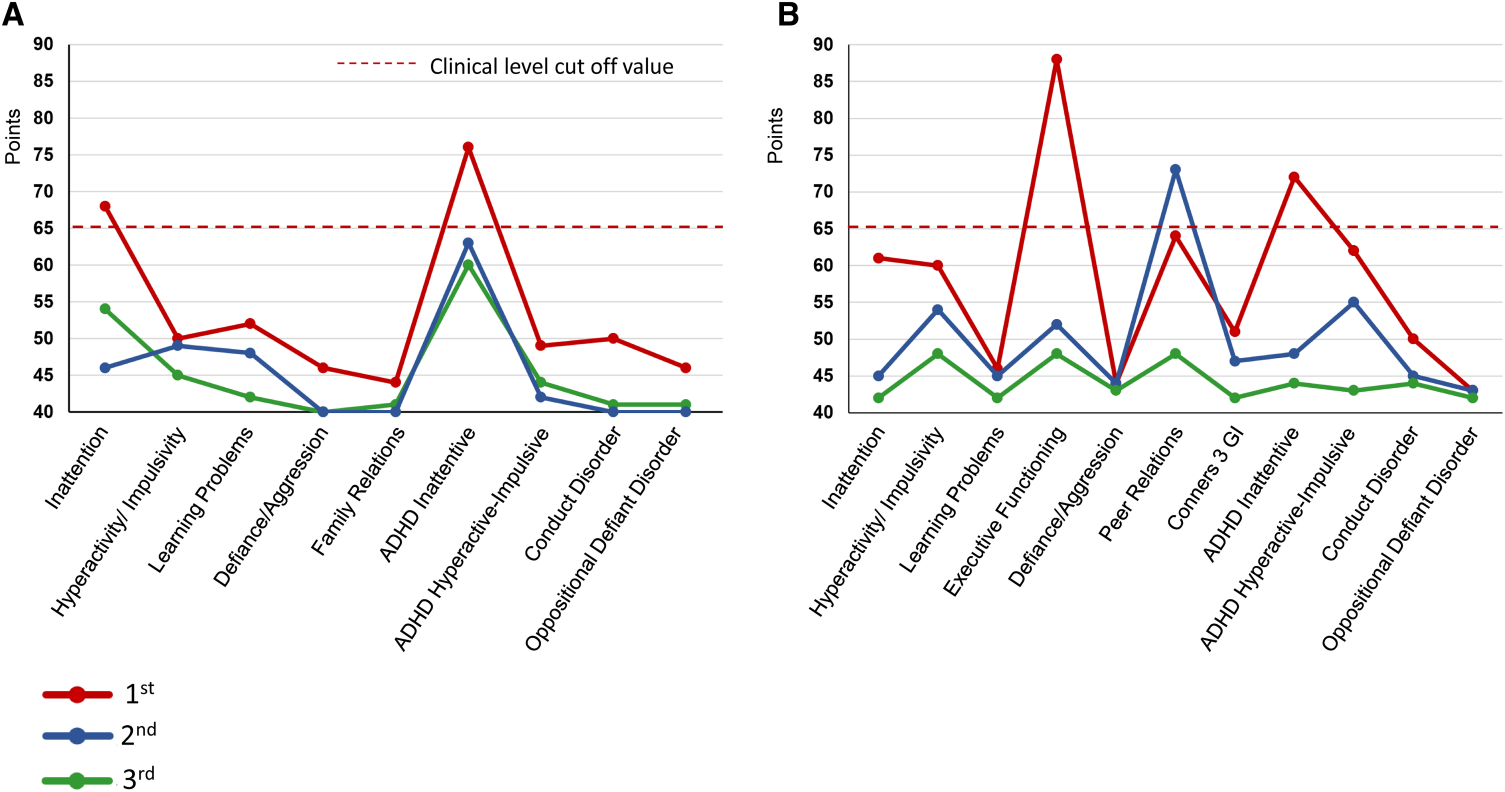
Changes in conners 3 scores during the Course of treatment. (**A**) Conners 3-Self Report (**B**) Conners 3-Parent. ADHD, Attention-Deficit/Hyperactivity Disorder; GI, Global Index.

During the first visit, the patient entered the examination room and, without greeting her physician, abruptly picked up a pain questionnaire from the desk and started responding to it. The patient turned on the swivel chair on which she sat, shook her legs, and often interrupted SK. She had already been diagnosed with ASD, but SK suspected that she may have comorbid ADHD. Thus, an ADHD symptom assessment was conducted from day 1–82, which confirmed the diagnosis. ADHD symptoms were assessed using the Conners 3-self-report and -parent assessment (22), completed by the patient and her mother, respectively (Figure 3). According to the results, her subscale scores exceeded 65 points and her ADHD symptoms were at the clinical psychiatric level (Figure 3). She was diagnosed with combined-type ADHD and satisfied 9/9 DSM-5 ADHD diagnostic criteria for inattention and 6/9 criteria for hyperactivity-impulsivity.

### 3.2. Therapeutic interventions and outcomes

**Figure 4:**
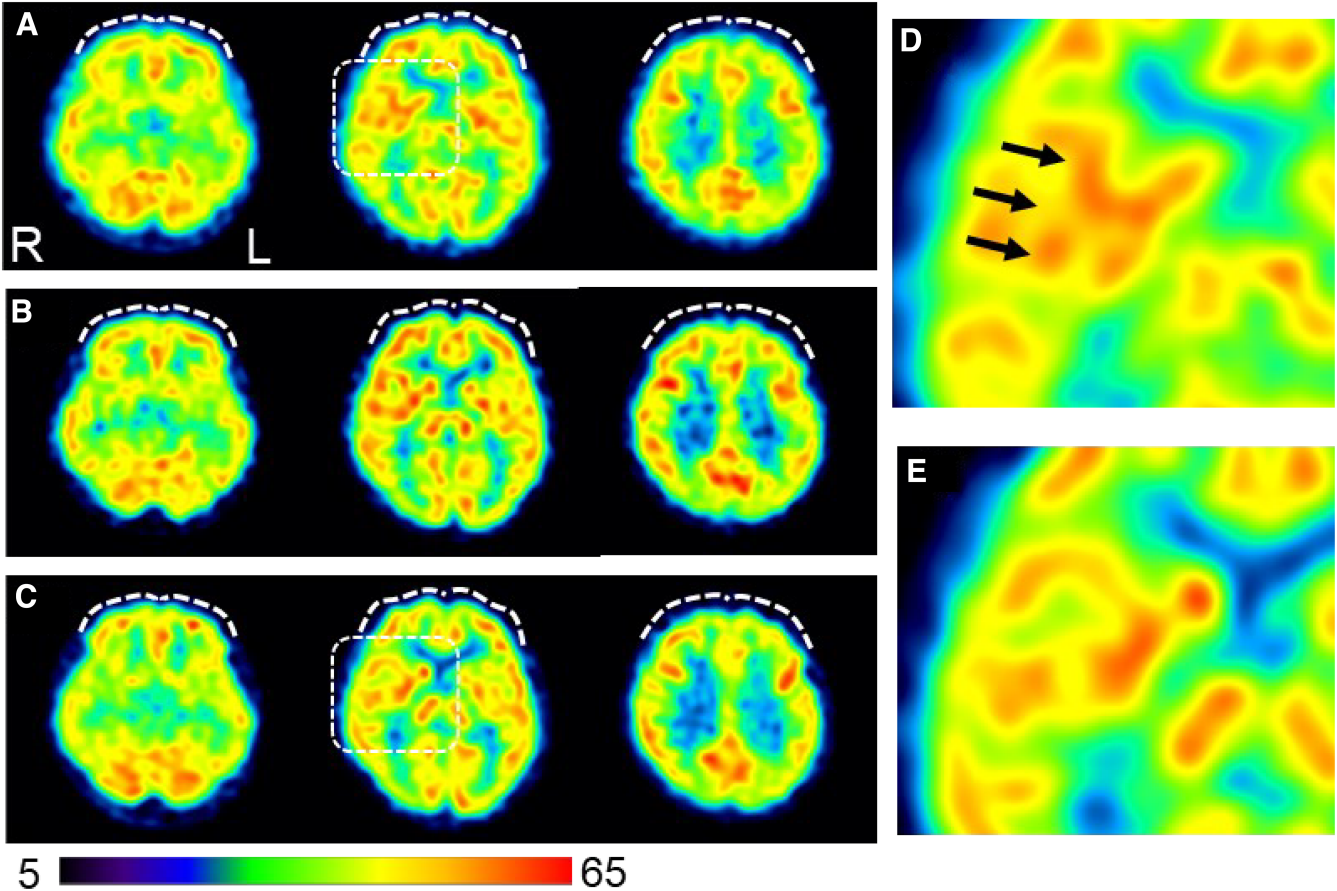
Changes in cerebral blood flow during the course of treatment. (**A**) Brain perfusion single-photon emission computed tomography images obtained on day 96 before initiating ADHD medications (**B**) Pain-free status on day 292 under administration of atomoxetine (50 mg/day). (**C**) A persistent pain-free status on day 1,503 is achieved with a combination of methylphenidate (18 mg/day) and guanfacine (4 mg/day). The three axial images from left to right indicate the inferior frontal, basal ganglia, and frontoparietal levels. The squared area on the axial images of the basal ganglia level in (**A**) and (**C**) are expanded in (**D**) and (**E**), respectively, to show a magnified view of the insular cortex area. The voxel values are normalized by the average counts-per-voxel with the cerebellum being 50; the color bar represents from 5 to 65. Mild hypoperfusion in the bilateral prefrontal cortices in (**A**) are slightly increased in (**B**) and returned to a similar level as (**A**) in (**C**) (white dashed curves). The insular cortex is identified in (**D**) (arrows), but not in (**E**), suggesting that the relative hyperperfusion in (**D**) is reduced in (**E**) The mean normalized voxel values for each image from (**A**) to (**C**) are as follows: 48.4, 50.1, and 48.5 in the prefrontal region and 54.3, 53.6, and 52.4 in the insular cortex, respectively. ^99m^Tc- ethyl cysteinate dimer (ECD; Fujifilm RI Pharma, Tokyo, Japan) at a dose of 740 MBq is administered to the patient resting in a supine position in a quiet room with her eyes closed. Approximately 10 min after injection, SPECT is performed for 30 min with a triple-head gamma camera (GCA-9300R; Canon Medical Systems Corp, Tochigi, Japan). Voxel value normalization and calculation are performed using PMOD version 3.7 (PMOD Technologies Ltd., Zurich, Switzerland). SPECT: single-photon emission computed tomography; ADHD, attention deficit hyperactivity disorder.

On day 96, single-photon emission computed tomography (SPECT) was performed before treatment initiation. Mild hypoperfusion of the frontal lobe and relative hyperperfusion of the insular cortex were observed (Figure 4). After initiating MP at 18 mg/day, the patient stated, “I began to have periods of zero pain. Various miscellaneous thoughts ceased to arise, and my thoughts became as quiet as the surface of a lake, which I had never experienced before.” She was able to sit up for the renal pediatrician's examination, whereas earlier, she always lay in bed due to back pain. The pediatrician was also surprised, saying, “Her eye focus and facial expressions are completely different.” However, MP was replaced with atomoxetine (ATX) 50 mg/day on day 187 due to side-effects, viz. headache, constipation, dry mouth, hypertension, and tachycardia. Thereafter, her face became much more animated. As the blood pressure increased to about 140/100 mmHg with MP or ATX, candesartan dose was increased from 2 to 4 mg/day on day 208, which resulted in improvement of the blood pressure to about 118/84 mmHg at outpatient visit.

A second SPECT was performed on day 292. The patient informed that she had been administered candesartan by a pediatrician, but still had high blood pressure and pulse rate and tired easily. Therefore, considering the possible side-effects of ATX such as hypertension and tachycardia, ATX 50 mg/day was changed to GF 3 mg/day. The remission of pain was maintained, and she was able to pass her high school equivalency examination. However, switching to GF resulted in the side-effect of hypotension and the dose of candesartan was reduced from 4 mg/day to 2 mg/day on day 362. The mother, who listened to the patient playing the piano in the living room every day while she cooked, stated that she was surprised to hear each note stand out, and the music she heard every day was completely different. The acetaminophen dose was reduced to 600 mg/day and tramadol 75 mg/day was discontinued. By day 684, the pain NRS, EuroQoL-5 Dimension, HADS, and PCS scores improved and remitted beyond the MCID, and the Conners 3 scores were normalized, except for the Peer Relations score on the Conners 3-Parent.

Subsequently, because monotherapy with GF 3 mg/day lowered blood pressure and caused sleepiness and lethargy during the day, MP 18 mg/day was added again on day 1,076, which also increased her blood pressure, concentration, and English comprehension scores.

On day 1,139, the patient experienced a tonic clonic seizure during dinner. She visited our psychiatric epilepsy outpatient clinic, was diagnosed with focal epilepsy, and was administered lamotrigine 250 mg/day. Stress from taking English tests was thought to have triggered the seizures. After the epileptic seizure, pain, anxiety/depression, and poor concentration worsened, and she was unable to study for approximately two months.

Thereafter, the dosage of MP and GF were adjusted according to blood pressure fluctuations, and finally, the condition was stabilized at 18 mg/day MP, 4 mg/day GF, and 250 mg/day lamotrigine. Following completion of medication adjustment, a third SPECT was performed on day 1,503. All assessment scale scores were normalized.

On day 1,632, the day before the college entrance examination, she started experiencing headaches and yawning-like signs of epilepsy, which made her feel ill and increased her pain, anxiety, and depression. However, she did not have seizures and took all the entrance examinations. She was accepted by the philosophy department of her university. She expressed hope for the future, saying that she would like to obtain a teaching certificate and work to provide academic support to children with long-term illness, akin to herself.

## 4. Discussion

This case demonstrated three distinctive findings: (1) ADPKD can coexist with ADHD and ASD, (2) GF and MP can improve chronic pain in ADPKD, and (3) cerebral blood flow can improve during the treatment course.

To the best of our knowledge, this is the first report of ADPKD co-existing with ADHD and ASD. Both, the patient and her siblings were diagnosed with ASD by a child psychiatrist; however, ADHD was overlooked. Although ADHD is present in 40%–85% of children with ASD (23, 24), clinicians miss more than half of ADHD cases with comorbid ASD (23). However, if ADHD occurs in a patient with ASD, medications for ADHD are available. Consistent with the findings in this case, studies have reported that ADHD medications can significantly improve chronic pain (25–31). It has also been reported that ADHD is more likely to be associated with chronic pain (32–34). Therefore, when treating pain associated with ADPKD, screening and treatment for ADHD and ASD should be considered.

The ADHD medications, GF and MP, improved ADPKD-induced pain. Monotherapy with MP or ATX was effective for both ADHD symptoms and pain, but caused side-effects. Monotherapy with MP or ATX was inappropriate for treating this patient because elevated blood pressure is an exacerbating factor in ADPKD symptoms. GF did not increase blood pressure in this patient and was considered appropriate for the management of ADPKD. However, when administered alone, GF causes side-effects, such as hypotension, fatigue, and drowsiness; therefore, MP 18–36 mg/day was administered in combination with GF, and blood pressure was maintained within the recommended range (<110/75 mmHg) (35). Thus, it seems necessary to adjust the combination of GF, MP, and ATX when administering ADHD medications to patients with ADPKD. Previous studies on GF and MP combination therapy indicated significantly improved ADHD symptoms with MP and GF than with MP alone, and most adverse events were anticipated with mild to moderate severity (36, 37). In the present case, GF and MP were administered in combination; however, no serious adverse events were observed.

In this case, hyperperfusion of the insular cortex on pretreatment cerebral blood flow-SPECT was reduced all through the persistent pain-free state during ADHD medication therapy. Brain imaging studies using positron emission tomography (PET) have shown hyperperfusion of the insular cortex during pain stimulation (38). The insular cortex is a part of the pain matrix (39) and is considered a component of the affective and emotional processes of nociception due to its neuronal connections with the thalamus and amygdala (40). Furthermore, a PET study showed that the degree of tonic pain was positively correlated with regional glucose metabolism in the insular cortex (41). Therefore, insular hyperactivity may reflect chronic pain associated with the emotional experiences of severe pain. Moreover, frontal perfusion increased slightly during treatment with ATX, which has a stimulant effect on catecholamine transmission in the prefrontal cortex (42). As the insular cortex is also interrelated with the frontal lobe (40), the changes in cerebral blood flow-SPECT in this case suggest that ATX modulates the insular cortex activity through frontal lobe activation, which is maintained by GF and MP, leading to pain relief.

Mutations in *PKD1* were detected in this patient, which may have contributed to cyst formation in ADPKD. Polycystin-1, encoded by *PKD1*, forms complex ion channels and regulates intracellular Ca concentration (1). Polycystin-1 is widely distributed in tissues throughout the body, but is particularly abundant in the brain, where it makes an important contribution to neurodevelopment (43–45). Mutations in *PKD1* have been reported as a possible cause of epilepsy following a history of febrile seizures (45). This patient had a history of febrile seizures at 5 years of age and epilepsy at 18 years, consistent with a previous study (the younger brother also had a *PKD1* gene mutation, which caused epileptic seizures at 14 years of age). On day 1,139, when the patient's epilepsy was untreated, she experienced a tonic clonic seizure when she took an English certification examination, which aggravated her mental function and prevented her from studying for approximately two months. However, during the examination on day 1,594, one year after receiving anti-epileptic treatment, she successfully progressed through the three-month examination season without having a seizure and was accepted into college. The coexistence of febrile seizures and epilepsy should be noted in ADPKD, in which mutations in *PKD1* account for approximately 85%–90% of cases.

There were two limitations to this study. First, the epileptic seizure on day 1,139 occurred after the addition of MP on day 1,076; MP could have lowered the seizure threshold and induced the seizure (46). However, MP was not discontinued because the patient requested it to continue owing to its benefits (47). Second, the three evaluations of cerebral blood flow in this case followed changes during the period of 15–18 years of age, and the possibility that the changes were not caused by drug therapy, but rather reflected changes in blood flow distribution with growth, cannot be ruled out. This should be taken into account when generalizing the results of this study.

This case report showed that ADHD and ASD may coexist in patients with ADPKD, and that combination treatment with GF and MP can improve chronic back pain associated with ADPKD, while maintaining blood pressure within the appropriate range and improving cerebral blood flow. Although the diagnosis of ADHD comorbid with ASD is easily missed, screening for ADHD may be important for the treatment of ADPKD since ADHD medications, such as GF and MP, can be administered if comorbidity is confirmed. However, this is the only case report, describing an association between ADPKD and ADHD or ASD. Further studies are required to establish the relationship between these disorders and their treatment.

## 5. Patient perspective

Previously, the patient found it difficult to cope with being continuously told that the pain was a mental problem or that it should not hurt that much whenever she visited a hospital. However, after our intervention, the patient discovered that her pain was related to brain function impairments. The ADHD medication improved both her ADHD symptoms and pain. Her older and younger brothers also visited SK's outpatient clinic for ADHD assessment, and both were diagnosed with ADHD. The family's understanding of the siblings' ADHD symptoms enabled them to respond more appropriately to their behavior. The patient and her mother were grateful that the ADHD was discovered and treated at the hospital.

## Data availability statement

The original contributions presented in the study are included in the article/supplementary Material, further inquiries can be directed to the corresponding author.

## Ethics statement

The studies involving humans were approved by the Research Ethics Committee of Tokyo University Hospital (approval no. 3678). The studies were conducted in accordance with the local legislation and institutional requirements. Written informed consent was obtained from the patient and her parents for the publication of this case report and any potentially identifiable data included in this article.

## References

[1] Mochizuki T Tsuchiya K Nitta K. Autosomal dominant polycystic kidney disease: recent advances in pathogenesis and potential therapies. Clin Exp Nephrol. (2013) 17(3):317–26. 10.1007/s10157-012-0741-0 23192769

[2] Solazzo A Testa F Giovanella S Busutti M Furci L Carrera P The prevalence of autosomal dominant polycystic kidney disease (ADPKD): a meta-analysis of European literature and prevalence evaluation in the Italian province of Modena suggest that ADPKD is a rare and underdiagnosed condition. PLoS One. (2018) 13(1):e0190430. 10.1371/journal.pone.0190430 29338003 PMC5770025

[3] van Luijk F Gansevoort RT Blokzijl H Groen GJ de Haas RJ Leliveld AM Multidisciplinary management of chronic refractory pain in autosomal dominant polycystic kidney disease. Nephrol Dial Transplant. (2023) 38(3):618–29. 10.1093/ndt/gfac158 35512573 PMC9976741

[4] Shteinberg M Haq IJ Polineni D Davies JC. Cystic fibrosis. Lancet. (2021) 397(10290):2195–211. 10.1016/S0140-6736(20)32542-3 34090606

[5] Georgiopoulos AM Friedman D Porter EA Krasner A Kakarala SP Glaeser BK Screening for ADHD in adults with cystic fibrosis: prevalence, health-related quality of life, and adherence. J Cyst Fibros. (2018) 17(2):276–80. 10.1016/j.jcf.2017.08.011 28867260

[6] Cohen-Cymberknoh M Tanny T Breuer O Blau H Mussaffi H Kadosh D Attention deficit hyperactivity disorder symptoms in patients with cystic fibrosis. J Cyst Fibros. (2018) 17(2):281–5. 10.1016/j.jcf.2017.11.020 29269187

[7] Georgiopoulos AM Hua LL. The diagnosis and treatment of attention deficit-hyperactivity disorder in children and adolescents with cystic fibrosis: a retrospective study. Psychosomatics. (2011) 52(2):160–6. 10.1016/j.psym.2010.12.016 21397109

[8] Cohen-Cymberknoh M Dimand I Tanny T Blau H Mussaffi H Kadosh D The association between attention-deficit-hyperactivity-disorder (ADHD) symptoms and disease severity in people with cystic fibrosis (pwCF). J Cyst Fibros. (2023) 22(4):772–6. 10.1016/j.jcf.2023.04.004 37061352

[9] Spitzer N Legare TB Patel P Toselli N Livingston F. The prevalence and effect of comorbid cystic fibrosis and attention deficit hyperactivity disorders on hospitalizations: a retrospective analysis. Cureus. (2018) 10(7):e3048. 10.7759/cureus.3048 30397565 PMC6207276

[10] Skieterska K Rondou P Van Craenenbroeck K. Dopamine D4 receptor ubiquitination. Biochem Soc Trans. (2016) 44(2):601–5. 10.1042/BST20150281 27068976

[11] Lukacs GL Verkman AS. CFTR: folding, misfolding and correcting the *Δ*F508 conformational defect. Trends Mol Med. (2012) 18(2):81–91. 10.1016/j.molmed.2011.10.003 22138491 PMC3643519

[12] Harris PC Torres VE. *Polycystic kidney disease, autosomal dominant; 2002* [updated Sep 29 2022]. Available at: https://pubmed.ncbi.nlm.nih.gov/20301424/ (Cited October 13, 2023).

[13] Jensen MP Karoly P. Self-report scales and procedures for assessing pain in adults. In: Turk DC Melzack R, editors. Handbook of pain assessment. 3rd ed. New York: The Guilford Press (2011). p. 19–44.

[14] Salaffi F Stancati A Silvestri CA Ciapetti A Grassi W. Minimal clinically important changes in chronic musculoskeletal pain intensity measured on a numerical rating scale. Eur J Pain. (2004) 8(4):283–91. 10.1016/j.ejpain.2003.09.004 15207508

[15] Balestroni G Bertolotti G. EuroQol-5D (EQ-5D): an instrument for measuring quality of life. Monaldi Arch Chest Dis. (2012) 78(3):155–9. 10.4081/monaldi.2012.121 23614330

[16] Suzuki H Aono S Inoue S Imajo Y Nishida N Funaba M Clinically significant changes in pain along the pain intensity numerical rating scale in patients with chronic low back pain. PLoS One. (2020) 15(3):e0229228. 10.1371/journal.pone.0229228 32126108 PMC7053735

[17] Zigmond AS Snaith RP. The hospital anxiety and depression scale. Acta Psychiatr Scand. (1983) 67(6):361–70. 10.1111/j.1600-0447.1983.tb09716.x 6880820

[18] Bjelland I Dahl AA Haug TT Neckelmann D. The validity of the hospital anxiety and depression scale. An updated literature review. J Psychosom Res. (2002) 52(2):69–77. 10.1016/s0022-3999(01)00296-3 11832252

[19] Puhan MA Frey M Büchi S Schünemann HJ. The minimal important difference of the hospital anxiety and depression scale in patients with chronic obstructive pulmonary disease. Health Qual Life Outcomes. (2008) 6:46. 10.1186/1477-7525-6-46 18597689 PMC2459149

[20] Sullivan MJL Bishop SR Pivik J. The pain catastrophizing scale: development and validation. Psychol Assess. (1995) 7(4):524–32. 10.1037/1040-3590.7.4.524

[21] American Psychiatric Association. “Section II: Diagnostic criteria and codes”. diagnostic and statistical manual of mental disorders. 5th ed Arlington, VA: American Psychiatric Publishing (2013). 31–781.

[22] Conners CK. Conners. 3rd ed [manual]: Multi-health systems. North Tonawanda, NY: Minnesota Historical Society Assessments (2008).

[23] Antshel KM Russo N. Autism spectrum disorders and ADHD: overlapping phenomenology, diagnostic issues, and treatment considerations. Curr Psychiatry Rep. (2019) 21(5):34. 10.1007/s11920-019-1020-5 30903299

[24] Yoshida Y Uchiyama T. The clinical necessity for assessing attention deficit/hyperactivity disorder (AD/HD) symptoms in children with high-functioning pervasive developmental disorder (PDD). Eur Child Adolesc Psychiatry. (2004) 13(5):307–14. 10.1007/s00787-004-0391-1 15490278

[25] Kasahara S Takahashi K Matsudaira K Sato N Fukuda KI Toyofuku A Diagnosis and treatment of intractable idiopathic orofacial pain with attention-deficit/hyperactivity disorder. Sci Rep. (2023) 13(1):1678. 10.1038/s41598-023-28931-3 36717626 PMC9887013

[26] Kasahara S Niwa SI Matsudaira K Sato N Oka H Yamada Y. Attention-deficit/hyperactivity disorder and chronic pain. Psychosom Med. (2020) 82(3):346–7. 10.1097/PSY.0000000000000789 32251099

[27] Kasahara S Takao C Matsudaira K Sato N Tu TTH Niwa SI Case report: treatment of persistent atypical odontalgia with attention deficit hyperactivity disorder and autism spectrum disorder with risperidone and atomoxetine. Front Pain Res (Lausanne). (2022) 3:926946. 10.3389/fpain.2022.926946 35935670 PMC9353025

[28] Kasahara S Kato Y Takahashi M Matsudaira K Sato N Niwa SI Case report: remission of chronic low back pain and oral dysesthesia comorbid with attention deficit/hyperactivity disorder by treatment with atomoxetine and pramipexole. Front Pain Res (Lausanne). (2023) 4:1159134. 10.3389/fpain.2023.1159134 37342213 PMC10277465

[29] Kasahara S Takahashi M Morita T Matsudaira K Sato N Momose T Case report: atomoxetine improves chronic pain with comorbid post-traumatic stress disorder and attention deficit hyperactivity disorder. Front Psychiatry. (2023) 14:1221694. 10.3389/fpsyt.2023.1221694 37608999 PMC10441107

[30] Kasahara S Kato Y Takahashi K Matsudaira K Sato N Fukuda KI Improvement in persistent idiopathic facial pain with comorbid ADHD using the combination of a dopamine system stabilizer and psychostimulant: a case report. Clin Case Rep. (2023) 11(6):e7552. 10.1002/ccr3.7552 37346882 PMC10279936

[31] Zain E Sugimoto A Egawa J Someya T. Case report: methylphenidate improved chronic pain in an adult patient with attention deficit hyperactivity disorder. Front Psychiatry. (2023) 14:1091399. 10.3389/fpsyt.2023.1091399 36970292 PMC10038200

[32] Kasahara S Matsudaira K Sato N Niwa SI. Attention-Deficit/hyperactivity disorder and centralized pain: a review of the case of john F. Kennedy. Clin Case Rep. (2022) 10(10):e6422. 10.1002/ccr3.6422 36245472 PMC9547351

[33] Kasahara S Niwa SI Matsudaira K Sato N Oka H Fujii T High attention-deficit/hyperactivity disorder scale scores among patients with persistent chronic nonspecific low back pain. Pain Phys. (2021) 24(3):E299–307. 10.36076/ppj.2021/24/E299 33988951

[34] Kasahara S Matsudaira K Sato N Niwa SI. Pain and attention-deficit/hyperactivity disorder: the case of margaret mitchell. Psychosom Med. (2021) 83(5):492–3. 10.1097/PSY.0000000000000947 33883539 PMC8189430

[35] Gimpel C Bergmann C Bockenhauer D Breysem L Cadnapaphornchai MA Cetiner M International consensus statement on the diagnosis and management of autosomal dominant polycystic kidney disease in children and young people. Nat Rev Nephrol. (2019) 15(11):713–26. 10.1038/s41581-019-0155-2 31118499 PMC7136168

[36] McCracken JT McGough JJ Loo SK Levitt J Del’Homme M Cowen J Combined stimulant and guanfacine administration in attention-deficit/hyperactivity disorder: a controlled, comparative study. J Am Acad Child Adolesc Psychiatry. (2016) 55(8):657–666.e1. 10.1016/j.jaac.2016.05.015 27453079 PMC4976782

[37] Wilens TE Bukstein O Brams M Cutler AJ Childress A Rugino T A controlled trial of extended-release guanfacine and psychostimulants for attention-deficit/hyperactivity disorder. J Am Acad Child Adolesc Psychiatry. (2012) 51(1):74–85.e2. 10.1016/j.jaac.2011.10.012 22176941

[38] Apkarian AV Bushnell MC Treede RD Zubieta JK. Human brain mechanisms of pain perception and regulation in health and disease. Eur J Pain. (2005) 9(4):463–84. 10.1016/j.ejpain.2004.11.001 15979027

[39] Legrain V Iannetti GD Plaghki L Mouraux A. The pain matrix reloaded: a salience detection system for the body. Prog Neurobiol. (2011) 93(1):111–24. 10.1016/j.pneurobio.2010.10.005 21040755

[40] Augustine JR. Circuitry and functional aspects of the insular lobe in primates including humans. Brain Res Brain Res Rev. (1996) 22(3):229–44. 10.1016/s0165-0173(96)00011-2 8957561

[41] Schreckenberger M Siessmeier T Viertmann A Landvogt C Buchholz HG Rolke R The unpleasantness of tonic pain is encoded by the insular cortex. Neurology. (2005) 64(7):1175–83. 10.1212/01.WNL.0000156353.17305.52 15824343

[42] Robbins TW Arnsten AFT. The neuropsychopharmacology of fronto-executive function: monoaminergic modulation. Annu Rev Neurosci. (2009) 32:267–87. 10.1146/annurev.neuro.051508.135535 19555290 PMC2863127

[43] Fagerberg L Hallström BM Oksvold P Kampf C Djureinovic D Odeberg J Analysis of the human tissue-specific expression by genome-wide integration of transcriptomics and antibody-based proteomics. Mol Cell Proteomics. (2014) 13(2):397–406. 10.1074/mcp.M113.035600 24309898 PMC3916642

[44] Stokely ME Hwang SY Hwang JY Fan B King MA Inokuchi K Polycystin-1 can interact with homer 1/vesl-1 in postnatal hippocampal neurons. J Neurosci Res. (2006) 84(8):1727–37. 10.1002/jnr.21076 17016857

[45] Wang JY Wang J Lu XG Song W Luo S Zou DF Recessive PKD1 mutations are associated with febrile seizures and epilepsy with antecedent febrile seizures and the genotype-phenotype correlation. Front Mol Neurosci. (2022) 15:861159. 10.3389/fnmol.2022.861159 35620448 PMC9128595

[46] Stahl SM. 82. Methylphenidate (d, l). In: Stahl SM, editors. Prescriber’s guide: Stahl’s essential psychopharmacology. Cambridge, UK: Cambridge University Press (2020). p. 487–94.

[47] Brikell I Chen Q Kuja-Halkola R D’Onofrio BM Wiggs KK Lichtenstein P Medication treatment for attention-deficit/hyperactivity disorder and the risk of acute seizures in individuals with epilepsy. Epilepsia. (2019) 60(2):284–93. 10.1111/epi.14640 30682219 PMC6365170

